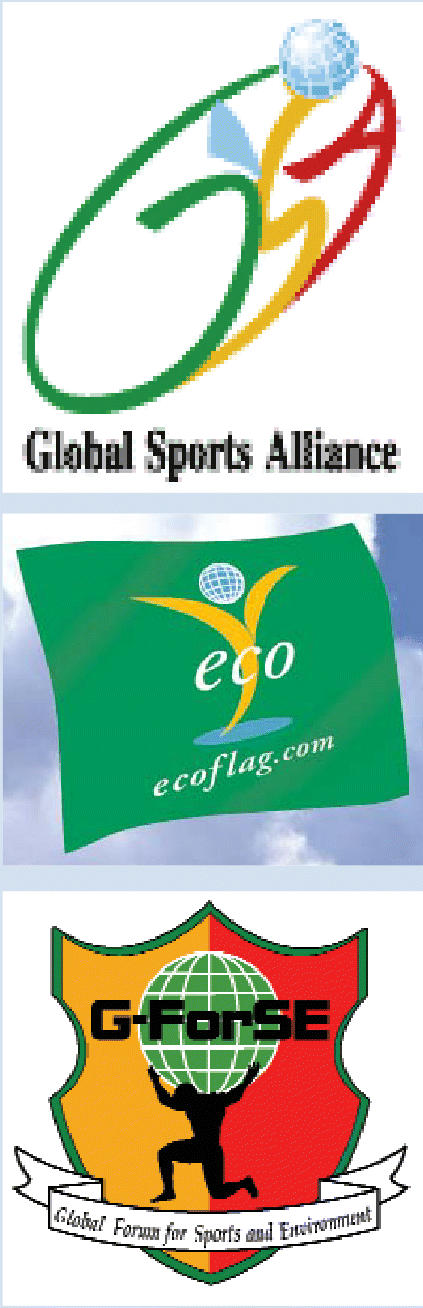# EHPnet: Global Sports Alliance

**Published:** 2006-05

**Authors:** Erin E. Dooley

Sports speak a universal language, bridging class, nationality, and religion around the world. Many sports figures are better known than movie stars or prominent politicians. To capitalize on the importance of sports to billions of people around the world, the Global Sports Alliance (GSA) was formed in 1999 to serve as an international network of sports enthusiasts who care about the environment. The English version of the GSA website, available at http://www.gsa.or.jp/en/index.html, describes the work of this group.

From the GSA homepage, visitors can access information on the alliance’s Ecoflag and Sports-eco.net projects. The Ecoflag, created by the GSA with the support of the UN Environment Programme (UNEP), is flown at sporting events around the world to symbolize the commitment of sporting enthusiasts to preserving the environment. Another component of Ecoflag is RECYCL’art, a movement to create works of art from used sports equipment, including balls, rackets, and shoes. The RECYCL’art website features a virtual gallery of such artwork. Sports-eco.net focuses on promoting the recycling of sports equipment. One alliance program collects used tennis balls and sends them to schools to put on the legs of school furniture to reduce noise in classrooms.

Another GSA project is the Global Forum for Sports and the Environment (G-ForSE), an archive of environmental action in sports from around the world. From a pull-down menu on the G-ForSE homepage, visitors can find information on how sports participants can protect the environment, as well as reviews of eco-friendly sporting goods such as battery-assisted bicycles, biodegradable fishing line, solar battery rechargers, and a portable ultraviolet measuring device.

As part of G-ForSe, the GSA sponsors Dream Camps in collaboration with UNEP, where children and teenagers are taught not only to play soccer and tennis, but also to be good environmental stewards. Camp activities include recycling and tree-planting projects. To date the camps have only taken place in Kenya, but the GSA is looking for other camp locations and organizers.

Through G-ForSE, the GSA also organizes global forums where world sport federation representatives, sporting goods manufacturers, athletes, and others join to discuss how the sports industry can bring environmental issues to the awareness of the global population and how to integrate sustainable practices into the industry itself. In July 2005, the Sports Summit for the Environment, held in Aichi, Japan, highlighted grassroots environmental initiatives through sports. Participants at the summit drew up the Joint Declaration on Sports and the Environment, which calls on the sports industry to become a partner in promoting sustainable development.

## Figures and Tables

**Figure:**